# Coronary microvascular dysfunction in patients undergoing transcatheter aortic valve implantation: A systematic review and meta-analysis

**DOI:** 10.1371/journal.pone.0360374

**Published:** 2026-10-15

**Authors:** Maethas Panyawongkhanti, Peerawat Chintrakulchai, Chonthicha Tanking, Wongsakorn Luangphiphat

**Affiliations:** 1 Princess Srisavangavadhana Faculty of Medicine, Chulabhorn Royal Academy, Bangkok, Thailand; 2 Chulabhorn Hospital, Chulabhorn Royal Academy, Bangkok, Thailand; Mount Sinai Hospital, UNITED STATES OF AMERICA

## Abstract

**Background:**

Coronary microvascular dysfunction (CMD) is a complication of severe aortic stenosis. Its prevalence after transcatheter aortic valve implantation (TAVI) and prognostic significance are understudied, as are the post-TAVI trajectories of microvascular indices.

**Aims:**

The aims of this study were to determine the pooled prevalence of CMD, the influence of the diagnostic modality, the post-TAVI coronary flow reserve (CFR) and microvascular resistance changes, and the prognostic impact of baseline CMD on major adverse cardiovascular events (MACE) in patients with severe aortic stenosis undergoing TAVI.

**Methods:**

Five databases were searched from January 2010 to December 2025 for studies involving adults with severe aortic stenosis undergoing TAVI in which CMD was assessed by wire-based thermodilution, angiography-derived indices, Doppler measurements, or stress cardiac magnetic resonance. The risk of bias and certainty of evidence were assessed using the Newcastle–Ottawa Scale and GRADE, respectively. Random-effects meta-analyses (DerSimonian–Laird) pooled CMD prevalence (stratified by modality), CFR change (mean difference; before–after design), and prognostic hazard ratios (HRs). Change in microvascular resistance was synthesized narratively. The protocol was prospectively registered (PROSPERO: CRD42025143775) and reported per the PRISMA 2020 guidance.

**Results:**

Eleven primary studies were analyzed, 10 of which (n = 1,212) contributed to the prevalence analyses. An additional study (n = 126) was included in the sensitivity analyses only. CMD prevalence was 46.8% (95% confidence interval [CI] [34.0, 59.9]; I^2^ = 72%) and 38.4% (95% CI [35.3, 41.7]; I^2^ = 0%) when assessed using wire-based thermodilution (k = 6, n = 324) and angiographic (k = 4, n = 888) indices, respectively. CFR increased post-TAVI by a pooled mean difference of +0.49 (95% CI [+0.27, + 0.71]; I^2^ = 72%; k = 5, n = 103 paired observations). Reductions in microvascular resistance were confined to pressure-to-velocity and reserve ratio indices, and absolute minimal microvascular resistance was unchanged (k = 4, n = 85). The HR for MACE was 3.22 (95% CI [0.98, 10.60]; I^2^ = 78%; k = 2, n = 585). Substantial heterogeneity reflected non-equivalent CMD definitions.

**Conclusion:**

The prevalence of CMD pre-TAVI is influenced by the diagnostic method. CFR tends to improve after TAVI, likely due to reduced resting demand rather than structural repair. The CMD–MACE association should be viewed as hypothesis-generating due to limited studies, varied CMD definitions, and incompatible HR calculations. Prospective multicenter studies with standardized CMD definitions are required.

## 1. Introduction

Severe aortic stenosis imposes a chronic pressure overload on the left ventricle that drives compensatory and maladaptive changes, including left ventricular hypertrophy, increased extravascular compressive forces, a reduction in the diastolic perfusion time, and altered coronary hemodynamics [1,2]. In addition to affecting the epicardial coronary physiology, these changes impact the coronary microcirculation, where reduced capillary density, perivascular fibrosis, and impaired vasodilatory reserve are collectively defined as coronary microvascular dysfunction (CMD) [2,3].

CMD has been associated with aortic stenosis for more than two decades [2,3]; however, the field has changed significantly over the past five years due to the development and validation of operator-independent quantitative indices. These include the continuous thermodilution-derived absolute coronary flow and microvascular resistance reserve (MRR) [4], the index of microcirculatory resistance (IMR), and angiography-derived surrogates, such as angiographic microvascular resistance (AMR), resting AMR (AMRr), and angiography-derived IMR (IMRangio) [5–8]. These tools allow systematic evaluation of CMD during the routine workup of patients undergoing transcatheter aortic valve implantation (TAVI), and their use has rapidly expanded the literature.

Despite these recent technological advances, three uncertainties hinder clinical interpretation. First, the prevalence of CMD varies widely across studies that use different indices and cut-points (15% to > 70%) [4,5,9,10], raising the question of whether CMD after TAVI is biologically meaningful. Second, while several studies have reported an improvement in the coronary flow reserve (CFR) after TAVI [10–12], this could reflect either structural microvascular recovery or a reduction in the resting myocardial demand following afterload relief—a mechanistic distinction with important clinical implications [12,13]. Third, baseline CMD has been associated with post-TAVI adverse outcomes. However, current prognostic estimates are based on the use of heterogeneous definitions, single-center cohorts, and different methodologies to calculate hazard ratios (HRs) [5,6,8].

Thus, we conducted a systematic review and meta-analysis with three prespecified objectives: to estimate the prevalence of CMD in patients with severe aortic stenosis undergoing TAVI, stratified by diagnostic modality; to quantify CFR and microvascular resistance changes after TAVI; and to synthesize the prognostic value of baseline CMD for major adverse cardiovascular events (MACE).

## 2. Methods

### 2.1 Protocol and registration

This systematic review and meta-analysis was prospectively registered in PROSPERO (CRD42025143775) and is reported in accordance with the PRISMA 2020 guidance [14]. The study was exempted from review by the Ethics Committee of Chulabhorn Royal Academy (IRB 077/2568, issued October 24, 2025).

### 2.2 Eligibility criteria

Studies that met the following criteria were eligible for inclusion: included patients aged ≥ 18 years who had severe aortic stenosis and were undergoing TAVI, included procedures performed using any device type or access route, and assessed CMD using validated invasive (CFR, IMR, MRR, AMR, AMRr, IMRangio, coronary flow velocity reserve (CFVR), or hyperemic microvascular resistance [HMR]) or imaging (stress cardiac magnetic resonance or positron emission tomography) indices. Prospective and retrospective studies were eligible, as were randomized trials, case–control studies, and cross-sectional studies. Studies that focused on pediatric populations or surgical-only cohorts were excluded, as were case reports with < 5 patients, non-human studies, and conference abstracts without the full text published.

### 2.3 Search strategy and study selection

Two reviewers (M.P. and W.L.) independently searched the Embase, CENTRAL, MEDLINE, Scopus, and Web of Science databases for articles published between January 1, 2010, and December 31, 2025, using a combination of MeSH and free-text terms (the full strings are provided in S1 Table). Two different reviewers independently screened the titles, abstracts, and full texts. Disagreements were resolved by consensus or by a third reviewer (C.T.). Inter-rater agreement was quantified using Cohen’s κ. Cohort overlap was assessed prospectively against five published criteria (S6 Table) and resolved before any meta-analytic pooling.

### 2.4 Risk of bias and certainty of evidence

To assess the risk of bias, the Newcastle–Ottawa Scale was used for cohort and case–control studies [15], the Cochrane RoB 2 tool was used for randomized trials, and Quality Assessment of Diagnostic Accuracy Studies-2 (QUADAS-2) was used for diagnostic-accuracy studies. The certainty of evidence for each pooled outcome was rated using the Grading of Recommendations, Assessment, Development, and Evaluations (GRADE) framework (Table 1) [16].

**Table 1:** Grading of Recommendations, Assessment, Development, and Evaluations (GRADE) summary of findings.

Outcome	Number of Studies (k)	Pooled Estimate	Certainty	Reasons for Downgrading
CMD prevalence (wire-based)	6	46.8% (34.0, 59.9)	⊕ Very Low	Inconsistency (I² = 72%), indirectness (heterogeneous thresholds), imprecision
CMD prevalence (angiographic)	4	38.4% (35.3, 41.7)	⊕⊕ Low	Indirectness (center-specific thresholds, lack of external validation)
Δ CFR (before vs. after TAVI)	5	MD + 0.49 (+0.27, + 0.71)	⊕⊕ Low	Indirectness (no concurrent control, cannot attribute change to TAVI alone)
Prognostic HR for MACE	2	HR 3.22 (0.98, 10.60)	⊕ Very Low	Inconsistency (I² = 78%), indirectness (different CMD definitions), imprecision (k = 2; CI crosses unity)

⊕⊕⊕⊕ High; ⊕⊕⊕ Moderate; ⊕⊕ Low; ⊕ Very Low. CFR = coronary flow reserve; CI = confidence interval; CMD = coronary microvascular dysfunction; HR = hazard ratio; MACE = major adverse cardiovascular events; MD = mean difference; TAVI = transcatheter aortic valve implantation

### 2.5 Data synthesis

All meta-analyses used a random-effects model (DerSimonian-Laird method) [17]. CMD prevalence was pooled on the logit scale, with a 0.5 continuity correction applied to studies with a prevalence of 0% or 100%. Given that the indices identify mechanistically and clinically distinct populations, CMD prevalence was stratified a priori by diagnostic modality (wire-based vs. angiography-derived). Mean differences between pre- and post-TAVI CFR data were calculated using a random-effects model. For three studies that reported the CFR as median [interquartile range] values, means and standard deviations were estimated using Wan et al.’s method [8] and flagged in the forest plot. Studies were eligible for the CFR-change pool if the cohort underwent TAVI only or TAVI-stratified data could be extracted, and paired before-and-after CFR measurements were reported for the same patients. Pre- to post-TAVI changes in microvascular resistance were not pooled but evaluated using narrative synthesis. The available studies reported indices that differ in physiological derivation and in the hemodynamic variables to which they are sensitive (HMR, MRR, and minimal microvascular resistance [Rμ]), and we concluded that a summary effect across them would lack a physiological referent. Individual study estimates were calculated as standardized mean differences (SMDs; Hedges’ g), with directionality harmonized so that negative values indicate reduced microvascular resistance after TAVI, and are presented grouped by index construct without a pooled estimate. Prognostic estimates were pooled as adjusted HRs on the log scale; only dichotomous adjusted estimates with their reported 95% confidence intervals (CIs) were eligible for the prognostic forest plot. Heterogeneity was assessed using the I^2^ statistic and τ^2^, which we interpreted as measures of statistical inconsistency only. With pools of k = 2–6, I^2^ is estimated imprecisely and biased toward zero when within-study variances are large [18,19]. The index used, the cut-point applied, the population enrolled, and the timing of assessment relative to the TAVI were appraised separately and qualitatively for every pool, and the pools that were judged to be clinically heterogeneous on these grounds were interpreted as such, irrespective of their I^2^ value. The prespecified sensitivity analyses are detailed in S9 Table.

## 3. Results

### 3.1 Study selection and characteristics

The PRISMA flow diagram presented in Fig 1 shows the study screening process. Among the 708 records identified, 389 underwent title and abstract screening after deduplication, and 31 were subjected to full-text review. Of the 12 publications that met the inclusion criteria, 11 contributed to the primary synthesis [4–12,20–22], Ten of these (n = 1,212) contributed to the prevalence pools, and the remaining study (Gallinoro et al. [20], n = 51) contributed to the CFR-change and resistance syntheses only. One further (n = 126) [21] was retained for the sensitivity analyses only, because it was an explicit pooled re-analysis of one of the included primary studies [4] plus two non-included cohorts. The characteristics of the included studies are summarized in Table 2, and the cohort overlap assessment is summarized in S6 Table [21].

**Table 2:** Characteristics of the included studies (k = 11 primary studies, plus one study included in the sensitivity analyses only [21]).

Study	Year	Design	Country	N (TAVI)	Age (median)	Female (%)	CMD method/threshold	Primary contribution
Scarsini et al. [5]	2024	Prosp. registry	Italy	250	83	53	IMRangio ≥ 30	Angio.-prev., prognostic (k = 2)
Scarsini et al. [4]	2024	Prosp. MC	Italy/ Belgium	134	82	54	MRR (lowest tertile)	Wire-prev., CFR change (n = 45)
Shi et al. [6]	2025	Prosp.	China	335	72	42	AMR > 250	Angio.-prev., prognostic (k = 2)
Shi et al. [8]	2025	Retro.	China	180	73	44	AMRr > 490	Angio.-prev., prognostic narrative only
Gallinoro et al. [20]	2024	Prosp.	Belgium	51	82	49	Q, Rμ, CFR (cont. thermo.)	CFR change (n = 20), resistance synthesis
Gallinoro et al. [21]	2025	Pooled (3 studies)	Italy/ Belgium	126	82	52	MRR ≤ 3	Sensitivity only (overlap)
Gutiérrez-Barrios et al. [11]	2024	Prosp.	Spain	23 (11 paired)	82	57	CFR, MRR (cont. thermo.)	Wire-prev., CFR change, resistance synthesis
Minten et al. [9]	2025	Prosp. MC (mixed AVR)	Belgium	116 enrolled (106 AVR: 68 TAVI/38 SAVR)	81	48	IMR > 25, MRR < 3, CFR	Wire-prev. (full cohort)
Wiegerinck et al. [10]	2015	Prosp.	Netherlands	27 (20 paired)	82	44	CFVR, HMR (Doppler)	Wire-prev., CFR change, resistance synthesis
Fezzi et al. [7]	2023	Retro. (blinded angio.)	Italy/ China	123	82	55	AMR, IMR ≥ 25 ref.	Angio.-prev.
Sabbah et al. [12]	2023	Prosp. (mixed AVR)	Denmark	17 TAVI (34 total)	79	41	CFR, Rμ (full cohort)	Wire-prev. (n = 17), resistance synthesis (n = 34)
Yones et al. [22]	2025	Prosp. pilot	United Kingdom of Great Britain and Northern Ireland	7 (10 vessels)	83	43	CFR, IMR, MVR (CMR)	Wire-prev., CFR change

angio-prev. = angiographic-based prevalence; AMR = angiographic microvascular resistance; AMRr = resting angiographic microvascular resistance; AVR = aortic valve replacement; CFR = coronary flow reserve; CFVR = coronary flow velocity reserve; CMD = coronary microvascular dysfunction; CMR = cardiovascular magnetic resonance; cont. thermo. = continuous thermodilution; HMR = hyperaemic microvascular resistance; IMR = index of microcirculatory resistance; IMRangio = angiography-derived index of microcirculatory resistance; MC = multicenter; MRR = microvascular resistance reserve; MVR = microvascular resistance; Prosp. = prospective; Q = absolute coronary flow; Retro. = retrospective; Rμ = absolute minimal microvascular resistance; SAVR = surgical aortic valve replacement; TAVI = transcatheter aortic valve implantation; TAVR = transcatheter aortic valve replacement; wire-prev. = wire-based prevalence

**Fig 1:**
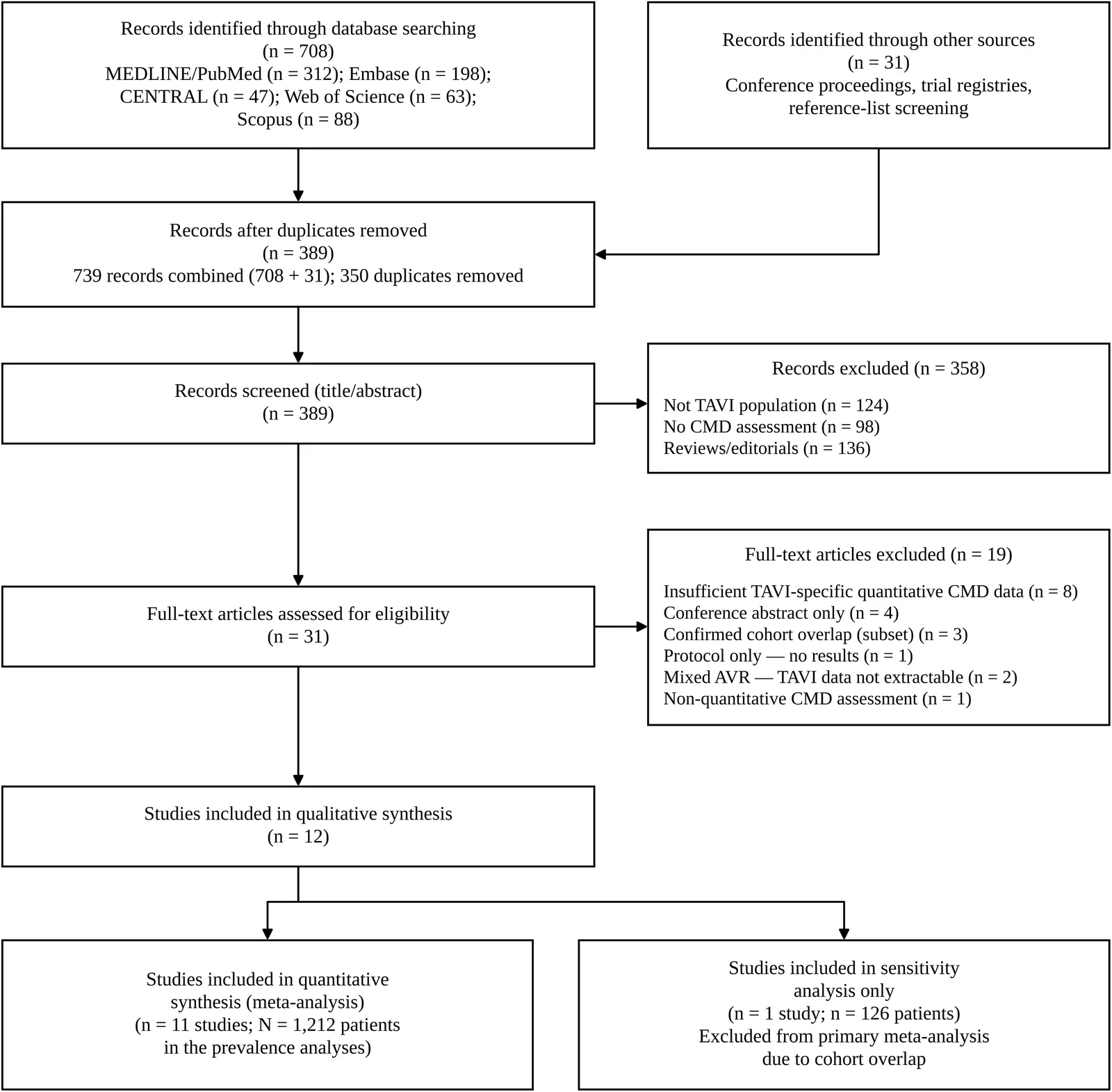
PRISMA 2020 flow diagram. Among the 708 records identified, 319 duplicates were removed. The remaining 389 unique records were screened, and 31 underwent full-text review. Twelve records were included in the analysis (11 in the primary synthesis, and Gallinoro et al. [21] was included in the sensitivity analyses). The reasons for exclusion during the full-text review are listed in S10 Table. Reporting follows the PRISMA 2020 guidelines [14].

Three sources of cohort overlap were prospectively identified and managed. According to Scarsini et al., their study published in 2023 [23] was based on a low-flow, low-gradient aortic stenosis subset of the multicenter cohort studied in their work published in 2024 [4]. Consequently, this study was excluded from all meta-analyses and is discussed narratively. Gallinoro et al. [21] pooled data from three prospective studies, including one by Scarsini et al. [4], and this was excluded from the primary forest plots and reserved for the sensitivity analyses, as prespecified. In the studies by Shi et al. [6,8], participants were recruited from the same Fuwai Hospital transcatheter aortic valve replacement (TAVR) registry; however, they enrolled different patient phenotypes (symptomatic vs. asymptomatic AS) and used different CMD metrics. Both studies were retained, with sensitivity testing (Table 3).

**Table 3:** Cohort overlap decisions.

Cohort Pair / Issue	Overlap Level	Decision and Rationale
Scarsini et al. [4] ↔ Gallinoro et al. [21]	High (explicit pooled re-analysis)	Gallinoro et al. [21]excluded from all primary forest plots, retained for sensitivity only.
Scarsini et al. [4] ↔ Scarsini et al. [23]	High (confirmed subset)	Scarsini et al. [23] excluded from all meta-analyses (LF LG-AS subset), discussed narratively only.
Shi et al. [6] ↔ Shi et al. [8]	Moderate (same Fuwai TAVR registry)	Both retained. Different patient phenotypes (symptomatic vs. asymptomatic), different CMD metrics.
Scarsini et al. [5] ↔ Scarsini et al. [4]	Low	Different modalities (IMRangio vs. MRR), different cohorts, confirmed independent.
Sabbah et al. [12] mixed AVR design	Design issue	TAVI subset (n = 17) used for prevalence pool. Excluded from CFR-change pool. Retained in narrative resistance synthesis (full cohort n = 34) because Rμ change is reported only for the full cohort and is consistent across AVR modes.
Minten et al. [9] mixed AVR design	Design issue,	Full cohort (n = 116) retained in wire-based prevalence pool because IMR-defined CMD prevalence is reported for combined cohort only.
Verona axis (Scarsini × Fezzi et al. [7])	Moderate	Possible Verona patient overlap. Sensitivity analysis excluding the study by Fezzi et al. [7] yielded angio. prevalence of 38.8% vs. primary 38.4% (negligible).

AVR = aortic valve replacement; CMD = coronary microvascular dysfunction; IMR = index of microcirculatory resistance; IMRangio = angiography-derived index of microcirculatory resistance; LFLG-AS=low-flow, low-gradient aortic stenosis; MRR = microvascular resistance reserve; Rμ = absolute minimal microvascular resistance; TAVI = transcatheter aortic valve implantation; TAVR = transcatheter aortic valve replacement

Two studies [9,12] enrolled mixed cohorts (i.e., patients underwent TAVI or surgical aortic valve replacement [SAVR]). Sabbah et al. [12] enrolled 34 patients (TAVI: n = 17, SAVR with bioprosthesis: n = 14, SAVR with mechanical prosthesis: n = 3), reported the change in CFR for the mixed aortic valve replacement (AVR) cohort only, and did not separately report the TAVI-specific paired CFR data. Thus, we used the TAVI-specific subset (n = 17) for the wire-based prevalence pool and excluded this study from the CFR-change forest plot because TAVI-only paired CFR data were not available. In the study by Sabbah et al. [12], the Rμ change for the full cohort was reported and showed no significant difference between the TAVI and SAVR subgroups. We retained it in the narrative resistance synthesis with this design limitation explicitly noted. Minten et al. [9] enrolled 116 patients, 106 of whom underwent AVR (TAVI: n = 68; SAVR: n = 38), and they reported the IMR-defined CMD prevalence for the combined AVR cohort only. The full cohort was retained in the wire-based prevalence pool, with the mixed AVR design flagged as a limitation.

### 3.2 CMD prevalence is modality-dependent

A comprehensive summary of all pooled estimates is presented in Table 4. The pooled prevalence of CMD differed substantially between the modalities. The wire-based pool (k = 6, n = 324) yielded a prevalence of 46.8% (95% CI [34.0, 59.9]; I² = 72%), whereas the angiography-derived pool (k = 4, n = 888) yielded a more consistent prevalence of 38.4% (95% CI [35.3, 41.7]; I² = 0%) (Table 5 and Fig 2).

**Table 4:** Summary of all pooled estimates.

Outcome	Number of Studies (k)	Number of participants (N)	Pooled Estimate (95% CI)	Heterogeneity	Interpretation
CMD prevalence (wire-based)	6	324	46.8% (34.0, 59.9)	I² = 72%	Substantial heterogeneity from threshold variation, stable sensitivity
CMD prevalence (angiographic)	4	888	38.4% (35.3, 41.7)	I² = 0%	Highly consistent across studies
Δ CFR (mean diff. between before and after TAVI)	5	103	+0.49 (+0.27, + 0.71)	I² = 72%, τ² = 0.044	Mechanism: reduced resting flow, not microvascular repair
Prognostic HR for MACE	2	585	3.22 (0.98, 10.60)	I² = 78%, τ² = 0.59	Exploratory only, CI crosses unity, substantial inter-study divergence

CFR = coronary flow reserve; CI = confidence interval; CMD = coronary microvascular dysfunction; HR = hazard ratio; MACE = major adverse cardiovascular events; MD = mean difference; TAVI = transcatheter aortic valve implantation

**Table 5:** Prevalence of coronary microvascular dysfunction by diagnostic modality (primary synthesis).

Modality	Number of Studies (k)	Number of participants (N)	Pooled prevalence (95% CI)	Heterogeneity	Notes
Wire-based thermodilution	6	324	46.8% (34.0, 59.9)	I^2^ = 72%, τ^2^ = 0.27	Includes the Sabbah et al. [12] TAVI subset (n = 17). Sensitivity excluding Yones et al. [22] (n = 7, all impaired): 43.9% (95% CI [32.5, 55.9]), I^2^ = 70%.
Angiographic	4	888	38.4% (35.3, 41.7)	I² = 0%	Highly consistent across IMRangio (Verona) and AMR/AMRr (Fuwai) cohorts despite different cut-points.
Combined (descriptive only)	10	1,212	Approximately 40% (descriptive)	I² = 55%	Modality-driven heterogeneity. Reported descriptively only, not endorsed as primary.

AMR = angiographic microvascular resistance; AMRr = resting angiographic microvascular resistance; CI = confidence interval; CMD = coronary microvascular dysfunction; IMR = index of microcirculatory resistance; IMRangio = angiography-derived index of microcirculatory resistance; TAVI = transcatheter aortic valve implantation

**Fig 2:**
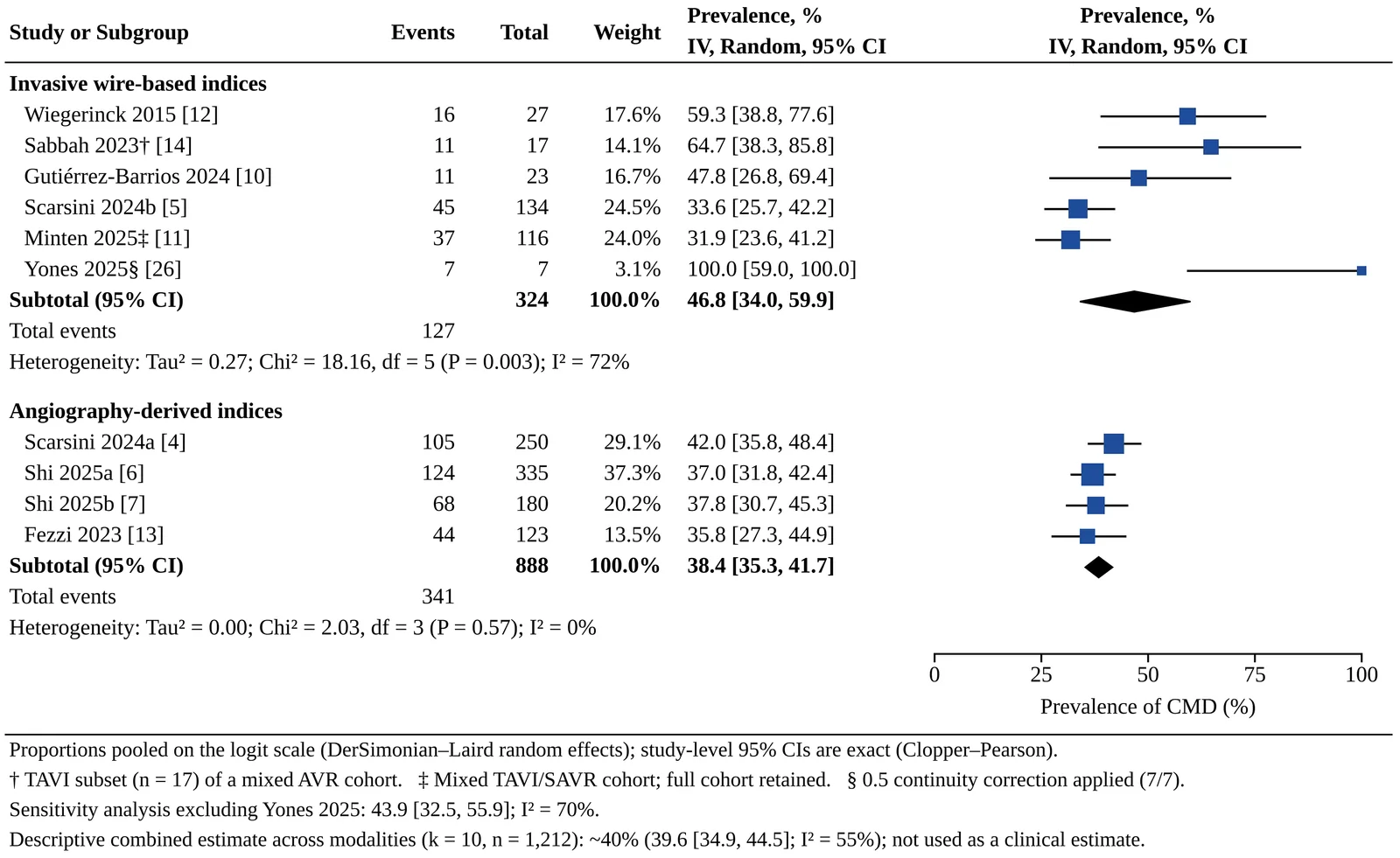
Prevalence of coronary microvascular dysfunction (CMD) by diagnostic modality (random-effects forest plot). Wire-based pool (k = 6, n = 324): pooled prevalence = 46.8% (95% confidence interval [CI] [34.0, 59.9]; I² = 72%). Angiographic pool (k = 4, n = 888): pooled prevalence = 38.4% (95% CI [35.3, 41.7]; I² = 0%). The combined pool of 1,212 patients yielded a descriptive estimate only due to modality-driven heterogeneity. Random-effects model, DerSimonian–Laird method [2]. CI = confidence interval; CMD = coronary microvascular dysfunction; k = number of studies; n = sample size.

The wire-based pool included the studies by Wiegerinck et al. [10] (16/27 = 59.3% by CFR < 2.0), Sabbah et al. [12] (TAVI subset, 11/17 = 64.7% by CFR < 2.5), Gutiérrez-Barrios et al. [11] (11/23 = 47.8% by CFR < 2.0), Scarsini et al. [4] (45/134 = 33.6% by lowest MRR tertile), Minten et al. [9] (37/116 = 31.9% by IMR > 25; full mixed AVR cohort retained), and Yones et al. [22] (7/7 = 100% by CFR < 2.5 + IMR > 25; all patients had concomitant coronary artery disease). Excluding the study by Yones et al. [22] lowered the pooled prevalence (43.9%, 95% CI [32.5, 55.9]) but did not materially reduce heterogeneity (I^2^ = 70%; S9 Table). The angiographic pool included the studies by Scarsini et al. [5] (105/250 = 42.0% by IMRangio ≥ 30), Shi et al. [8] (124/335 = 37.0% by AMR > 250), Shi et al. [7] (68/180 = 37.8% by AMRr > 490), and Fezzi et al. [7] (44/123 = 35.8% by IMR ≥ 25 AMR-validated reference).

The wire-based studies enroll smaller, more selected cohorts and use thresholds (CFR < 2.0–2.5, IMR > 25, lowest-tertile MRR) calibrated to detect functional impairment, whereas angiographic studies use larger, more representative cohorts and center-specific cut-points (IMRangio≥30, AMR > 250, AMRr > 490) [4–8]. A pooled combined estimate is reported descriptively only (Table 5), because the modality-driven heterogeneity precludes its endorsement as a clinically meaningful single estimate.

### 3.3 CFR rises after TAVI, primarily through reduced demand at rest

The post-TAVI CFR was significantly higher than the pre-TAVI CFR. The pooled random-effects mean difference was + 0.49 (95% CI [+0.27, + 0.71]; I^2^ = 72%; τ^2^ = 0.044; k = 5, n = 103 paired observations) (Fig 3). The studies that contributed to the pool were those by Wiegerinck et al. [10] (n = 20, mean difference [MD] = + 0.30 [+0.07, + 0.53]), Gutiérrez-Barrios et al. [11] (n = 11, MD = +0.60 [+0.39, + 0.81]), Gallinoro et al. [20] (n = 20, MD = +0.20 [−0.02, + 0.42]), Scarsini et al.’s [4] low-MRR subgroup (n = 45, MD = +0.65 [+0.28, + 1.02]; Wan et al.’s [24] method imputed), and Yones et al. [22] (n = 7, MD = +0.82 [+0.48, + 1.16]; Wan et al.’s [24] method imputed) (Table 6).

**Table 6:** Pooled estimates: Comparative analysis of microvascular indices before and after transcatheter aortic valve implantation.

Outcome	Number of Studies (k)	Number of participants (N)	Pooled estimate (95% CI)	Heterogeneity	Mechanistic interpretation
Δ CFR (mean difference)	5	103 paired	+0.49 (+0.27, + 0.71)	I² = 72%, τ² = 0.044	Driven by reduction in resting flow following afterload relief. Minimal microvascular resistance unchanged.

CFR = coronary flow reserve; CI = confidence interval; TAVI = transcatheter aortic valve implantation

**Fig 3:**
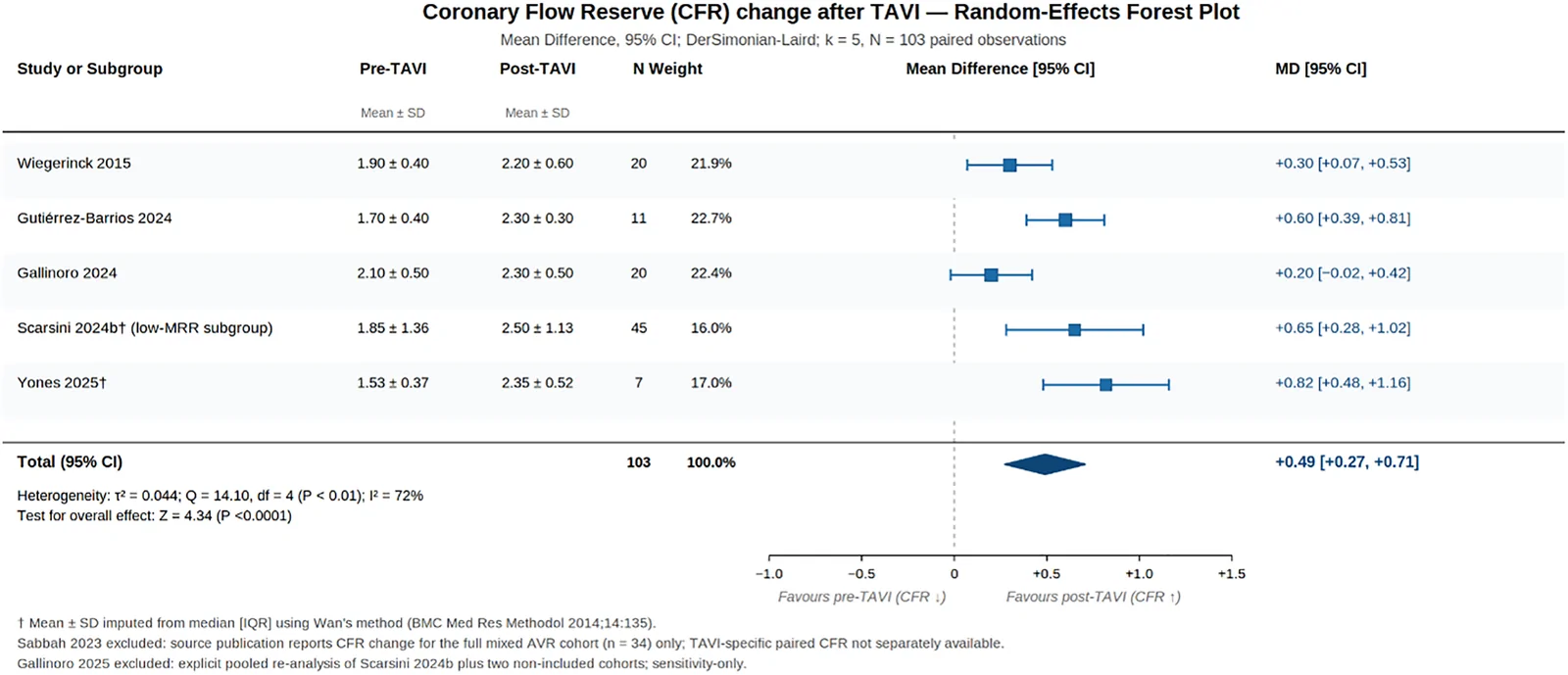
Forest plot for change in coronary flow reserve (CFR) after transcatheter aortic valve implantation (TAVI). Random-effects pooled mean difference: + 0.49 (95% confidence interval [+0.27, + 0.71]; I^2^ = 72%; τ^2^ = 0.044; k = 5, n = 103 paired observations). † indicates the studies for which Wan et al.’s [24] method was used to estimate the mean ± standard deviation from the median [interquartile range]. The study by Sabbah et al. [12] was excluded because the change in CFR was reported for the mixed cohort only and TAVI-specific paired CFR data were not separately reported. The study by Gallinoro et al. [21] was excluded because it pooled data from Scarsini et al. [21]. CFR = coronary flow reserve; CI = confidence interval; IQR = interquartile range; k = number of studies; MD = mean difference; n = sample size; SD = standard deviation; TAVI = transcatheter aortic valve implantation; τ^2^=between-study variance.

The study by Sabbah et al. [12] was excluded from this pool because it reported the CFR for the mixed AVR cohort only (median before = 2.5 [1.5–3.3], median after = 3.1 [2.2–5.1]; *p* = 0.005) and not for the TAVI-specific paired cohort only. Its inclusion would have introduced 17 SAVR patients into a TAVI-specific analysis. The study by Gallinoro et al. [21] was excluded because it pooled data from the study by Scarsini et al. [4]. Including this study in the sensitivity analysis resulted in a similar estimate (MD = +0.41 [+0.25, + 0.57]; S9 Table).

Regarding the mechanism underlying the improvement in CFR, among the pooled studies that measured absolute coronary flow, the rises in CFR and MRR reported by Gutiérrez-Barrios et al. [11] were driven by a reduction in resting flow, whereas Gallinoro et al. [20] observed no significant change in resting or hyperemic flow or in Rμ. Outside the pool, Sabbah et al. [12] (mixed AVR cohort) found that hyperemic flow and Rμ were unchanged and explicitly attributed the CFR rise to a fall in the demand at rest following afterload relief, consistent with reduced left ventricular stroke work [13]. Yones et al. [22] reached the same conclusion; using stress cardiovascular magnetic resonance (CMR) and computational vessel-specific modeling, they showed that the resting myocardial blood flow fell by 37% (3.0 ± 0.98 to 1.9 ± 0.7 mL/min/g; *p* = 0.033), while microvascular resistance was unchanged. Our findings support the mechanistic hypothesis of hemodynamic unloading rather than structural microvascular repair [13,25].

### 3.4 Microvascular resistance after TAVI: Structured synthesis without pooling

Pooled analysis of microvascular resistance changes after TAVI was precluded by the physiological differences between the indices measured. Instead, a narrative synthesis of four studies was performed (n = 85) (Fig 4). Wiegerinck et al. [10] (n = 20) observed a decline in HMR (SMD = −0.56 [−1.03, −0.09]), and Gutiérrez-Barrios et al. [11] (n = 11) reported an MRR change in the same direction that did not reach significance (MRR×−1; SMD = −0.49 [−1.12, + 0.13]). Direct measures of the absolute Rμ were largely unchanged in the studies by Gallinoro et al. [20] (n = 20; SMD = −0.18 [−0.62, + 0.26]) and Sabbah et al. [14] (n = 34; SMD = −0.20 [−0.53, + 0.14]). The study by Gallinoro et al. [21] was excluded due to cohort overlap. The study by Sabbah et al. [12] was retained in this synthesis, as the biological signal (unchanged Rμ) was consistent across its mixed-AVR cohort, although this mixed design was a limitation.

**Fig 4:**
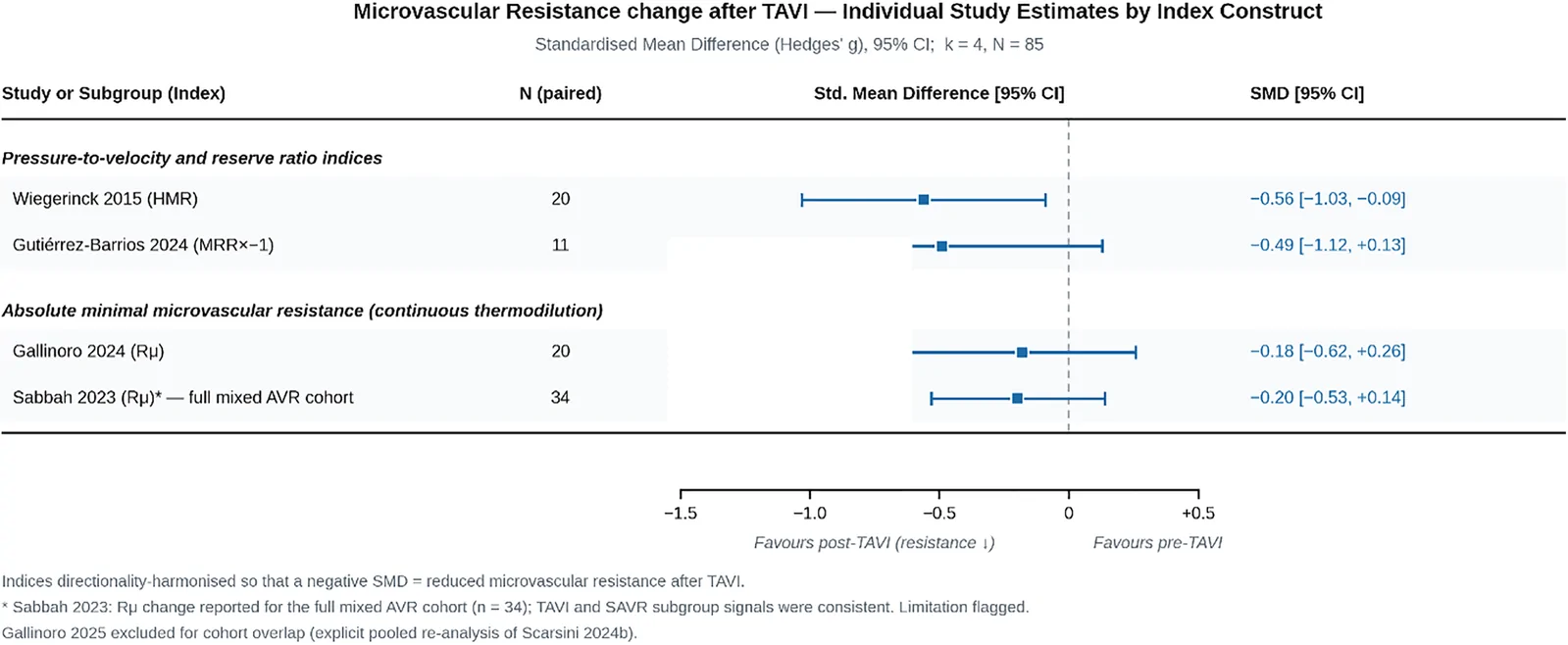
Change in microvascular resistance after transcatheter aortic valve implantation (TAVI). Individual study estimates are grouped by the index construct (Hedges’ g). Standardized mean differences with 95% confidence intervals for the four studies that reported paired measurements. Negative values indicate reduced microvascular resistance after TAVI. The study by Gallinoro et al.[21] was excluded due to cohort overlap. AVR = aortic valve replacement; HMR = hyperemic microvascular resistance; MRR = microvascular resistance reserve; N = sample size; Rμ = absolute minimal microvascular resistance; SMD = standardized mean difference; TAVI = transcatheter aortic valve implantation.

While HMR, MRR, and Rμ are physiologically related, they are not interchangeable, and they differ in their derivation, units, and sensitivity to hemodynamic variables. Hence, it is not appropriate to pool these indices. Rμ, the absolute measure of minimal microvascular resistance, is derived from continuous thermodilution. HMR is the ratio of distal pressure to hyperemic flow velocity. Given that reducing the transvalvular gradient raises distal coronary pressure and alters velocity, this index can shift without any change in true microvascular resistance. MRR is a reserve ratio normalized to resting flow and epicardial driving pressure and is therefore sensitive to the fall in resting myocardial demand that follows afterload relief. Expressing these measures as an SMD removes their units but does not reconcile the differences in their construction, and a summary effect across them would not correspond to any single measurable physiological quantity. Therefore, no pooled estimate is reported for this outcome. For transparency, an estimate obtained by combining all four studies is reported in S9 Table as part of the sensitivity analysis. The pool resulting from combining these four studies would show no statistical heterogeneity (I^2^ = 0%; τ^2^ = 0.000), which would indicate that the four standardized effect estimates of a small directional change are statistically compatible with one another. However, it would not indicate that HMR, MRR, and Rμ are interchangeable measures of microvascular status, nor that the underlying microvascular biology is homogeneous across the cohorts [10–12,20].

### 3.5 Exploratory prognostic association between CMD and MACE

Two studies reported dichotomous adjusted HRs for MACE in CMD-positive versus CMD-negative groups using the original study’s cut-points: Scarsini et al. [5] (n = 250; IMRangio ≥ 30; adjusted HR = 6.67, 95% CI [2.25, 19.78]) and Shi et al. [6] (n = 335; AMR > 250; HR = 1.94, 95% CI [1.34, 2.81]). The pooled exploratory HR was 3.22 (95% CI [0.98, 10.60]; I² = 78%; τ² = 0.59; n = 585) (Fig 5). The substantial heterogeneity reflected fundamental differences in the definition of CMD. Scarsini et al. [5] used IMRangio ≥ 30, a center-specific threshold derived in Verona, and Shi et al. [6] used AMR > 250, a Receiver Operating Characteristic (ROC)-optimized threshold from a single-center Chinese cohort. These definitions identify different patient populations, and it should not be assumed that they are clinically interchangeable. The wide CI, which crosses unity, mandates that this estimate be interpreted as hypothesis-generating only.

**Fig 5:**
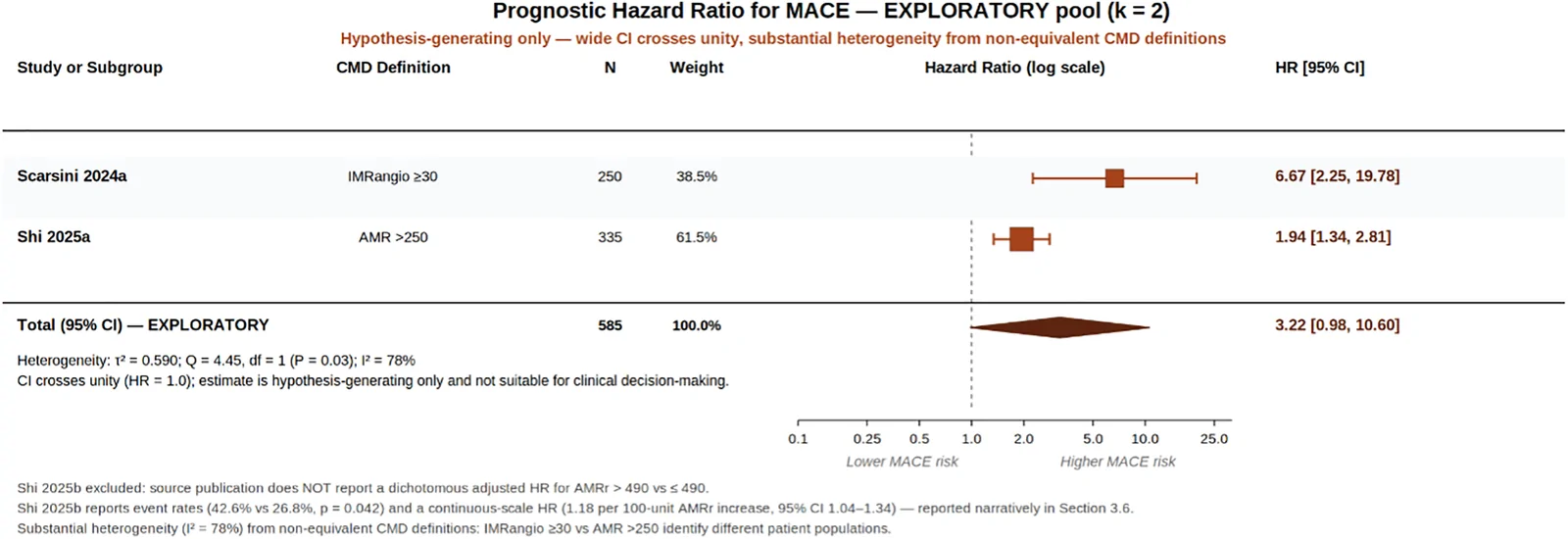
Forest plot for exploratory pooled hazard ratio (HR) for major adverse cardiovascular events (log scale). Random-effects pooled HR = 3.22 (95% confidence interval [0.98, 10.60]; I^2^ = 78%; τ^2^ = 0.59; k = 2, n = 585). The substantial heterogeneity reflected the use of fundamentally different definitions of coronary microvascular dysfunction (CMD). Scarsini et al. [5] used IMRangio ≥ 30 (HR: 6.67) and Shi et al. [6] used AMR > 250 (HR: 1.94). The study by Shi et al. [8] was excluded because it did not report a dichotomous adjusted HR for AMRr > 490 vs. ≤ 490; its event-rate finding (42.6% vs. 26.8%, *p* = 0.042) and continuous-scale HR (1.18 per 100-unit AMRr) are reported narratively in section 3.5. The pooled estimate should be regarded as exploratory only. AMR = angiographic microvascular resistance; AMRr = resting angiographic microvascular resistance; CI = confidence interval; CMD = coronary microvascular dysfunction; HR = hazard ratio; IMRangio = angiography-derived index of microcirculatory resistance; k = number of studies; MACE = major adverse cardiovascular events; N = sample size; n = sample size; τ^2^=between-study variance.

A third study [8] reported MACE outcomes in relation to AMRr (n = 180; asymptomatic/minimally symptomatic AS, single-center cohort, Fuwai Hospital). It detailed MACE rates by AMRr stratum (MACE in 42.6% of patients with AMRr > 490 vs. 26.8% of patients with AMRr ≤ 490; *p* = 0.042 by log-rank test) and a continuous-scale adjusted HR of 1.18 per 100-unit increase in AMRr (95% CI [1.04, 1.34]). However, a dichotomous adjusted HR for the AMRr > 490 versus AMRr ≤ 490 comparison was not reported. Deriving such a dichotomous estimate from Kaplan–Meier curves combined with the continuous estimate, in the absence of individual patient data, would entail compounding assumptions about the AMRr distribution and the local hazard at the cut-point that we judged methodologically unsound. Thus, Shi et al.’s [8] findings are reported narratively here, and this study was excluded from the prognostic forest plot (the methodological reasoning is detailed in S6 Table). Directionally, Shi et al.’s [8] results are consistent with an adverse prognostic association.

### 3.6 Sensitivity analyses

The results of the prespecified sensitivity analyses (S9 Table) confirmed the robustness of the principal findings. The wire-based prevalence estimate was stable after adding the study by Gallinoro et al. [21] (46.0% vs. primary 46.8%) and removing the small cohort from the study by Yones et al. [22] (43.9%; I² = 70% vs. 72%). The angiographic prevalence estimate was unchanged by the removal of the study by Fezzi et al. [7] (38.8% vs. 38.4%), which had possible Verona patient overlap with the Scarsini et al. [4,5] cohorts. The CFR-change estimate was robust in the leave-one-out analysis. The exploratory prognostic estimate was sensitive to the removal of individual studies (HR = 1.94 with Shi et al. [6] alone; HR = 6.67 with Scarsini et al. [5] alone), reinforcing its hypothesis-generating status.

## 4. Discussion

### 4.1 Principal findings

There are four important clinical and biological findings of this systematic review and meta-analysis of 11 primary studies of patients undergoing TAVI for severe aortic stenosis (1,212 patients in the prevalence analyses). First, CMD prevalence is highly dependent on the diagnostic modality. It was approximately 47% when wire-based thermodilution indices were used and 38% when angiography-derived indices were utilized. While the angiographic pool results are more clinically generalizable, the wire-based pool results are more mechanistically informative. Second, CFR is approximately 0.5 units higher after TAVI. This increase is due to a reduction in the resting myocardial demand that results from afterload relief rather than an improvement in the Rμ, which was unchanged when directly measured [12,13,20]. Third, the measurement index influences the observed post-TAVI changes in microvascular resistance, and the utilized indices are not amenable to pooling. Reductions were confined to indices derived from pressure-to-velocity or reserve ratios, whereas absolute Rμ was unchanged in both studies in which it was measured directly. Finally, a higher level of baseline CMD is linked to a higher risk of MACE after TAVI. However, because the combined exploratory HR was based on only two adjusted dichotomous estimates that used noncomparable definitions, it is too unstable—both statistically and methodologically—to be useful for individual patient counseling [5,6,8].

### 4.2 Mechanism underlying CFR improvement after TAVI

The post-TAVI rise in the CFR was consistent across the five contributing studies. In addition, within the CFR-change pool, Rμ was measured directly only by Gallinoro et al. [20] and did not change after TAVI, and CMR-derived microvascular resistance was likewise unchanged in the study by Yones et al. [22]; Rμ was also unchanged in the mixed AVR cohort of Sabbah et al. [12], which was excluded from the pool. Together, these findings indicate that the post-TAVI rise in the CFR was driven by a reduction in resting flow following afterload relief. This is supported by Sabbah et al.’s [12] finding of a 39% indexed increase in hyperemic flow per gram of myocardium with unchanged absolute hyperemic flow, and Yones et al.’s [22] finding of a 37% reduction in resting myocardial blood flow via stress CMR. In terms of the clinical implications of this finding, a rise in the CFR after TAVI should not be interpreted as evidence of microcirculation repair [13,25].

### 4.3 Prognostic significance of baseline CMD

Both Scarsini et al. and Shi et al. [5,6] found that baseline CMD is an independent predictor of MACE after multivariable adjustment, and Shi et al. [8] extended this association to the asymptomatic AS population using event-rate analysis. However, the pooled HR of 3.22 and its wide CI that crossed unity reflected the heterogeneity of these findings. The sevenfold risk in the IMRangio-based Verona cohort and the twofold risk in the AMR-based Fuwai cohort cannot be combined into a stable summary estimate. We have flagged this as an exploratory and hypothesis-generating finding, and we caution against using the pooled point estimate to inform individual patient counseling. Nevertheless, across three cohorts that were analyzed using different CMD definitions, the direction of the association was consistent: patients with baseline CMD experienced more MACE [5,6,8,25,26].

### 4.4 Methodological choices and corrections

Several methodological choices made in this review warrant explanation. First, the study by Sabbah et al. [12] was excluded from the CFR-change forest plot. Including the mixed cohort would have introduced 17 SAVR patients into a TAVI-specific analysis. The TAVI-specific subset from Sabbah et al. [12] was retained in the wire-based prevalence pool, and the full-cohort Rμ data from this study were included in the resistance synthesis, with the design limitation flagged. Second, the study by Gallinoro et al. [21] was excluded from all primary forest plots because it pooled data from one of our included studies [4] and its inclusion would have led to double-counting. Third, the study by Shi et al. [8] was excluded from the prognostic forest plot because it reported only a continuous-scale HR and event-rate stratification, and not a dichotomous adjusted HR that compared AMRr > 490 and AMRr ≤ 490 data. Deriving such an estimate from Kaplan–Meier curves and the continuous HR without individual patient data would have required indefensible assumptions.

### 4.5 Clinical and research implications

Our findings have clinical and research implications. They indicate that there is an urgent need for a standardized definition of CMD in patients undergoing TAVI. CMD is present in approximately 40%–50% of patients undergoing TAVI, and the prevalence is influenced by the type of indices used. The use of different definitions, indices, and cut-points, which are calibrated to single centers, is the primary obstacle hindering the development of a stable prognostic estimate [3,27]. Our findings also suggest that resting flow, hyperemic flow, and Rμ must be measured separately to distinguish structural microvascular recovery from hemodynamic unloading [13,25]. It is not sufficient to measure CFR alone. Whether persistent post-TAVI CMD is amenable to therapy (e.g., the use of statins, Angiotensin-Converting Enzyme inhibitors (ACE-inhibitors)/Angiotensin Receptor Blockers (ARBs), beta-blockers, ranolazine, Sodium-glucose cotransporter 2 inhibitors (SGLT2 inhibitors) warrants further prospective study [3,27,28].

CMD present before TAVI is directionally associated with worse MACE; however, the heterogeneity in the cut-points and HR derivation across centers means that the magnitude of the association is currently unclear. Until a consensus definition is established, centers reporting CMD outcomes of TAVI should always state the index used, the threshold applied, and whether the cut-point was prespecified or determined post hoc. Without such information, the prevalence and risk estimates from different cohorts cannot be meaningfully compared.

### 4.6 Limitations

This study has several limitations. Most of the pools were small (k = 2–6) and contained heterogeneous diagnostic indices. There was no predominant CMD definition. There was overlap between several of the included cohorts (i.e., the cohorts drawn from the Verona–Aalst–Milan axis and those drawn from Fuwai Hospital), which may have inflated the apparent reproducibility [4,5,7,20,21]. Two of the included studies [9,12] enrolled mixed AVR cohorts, and this was addressed via stratified extraction or explicit declaration. The before–after design of the change-in-CFR and change-in-resistance pools precludes the establishment of causation between TAVI and the observed changes. The absence of contemporaneous control groups is a design limitation of published studies. The funnel-plot assessment of publication bias was not informative, given the small number of studies per pool. The included studies used different modalities to measure or derive the CFR, and this methodological heterogeneity may have affected the magnitude and comparability of the reported changes. The small number of contributing studies precluded a subgroup analysis stratified by CFR assessment modality, and the pooled CFR estimate should be interpreted accordingly. The low statistical heterogeneity in several of our pools should not be equated with clinical or biological homogeneity. For the microvascular resistance outcome, the use of distinct physiological constructs precluded pooling. The angiography-derived prevalence pool, which included data from studies that applied center-specific cut-points to different populations, had an I² value of 0%. These studies differed materially in their threshold, case mix, and timing of assessment. Because I² was estimated from very few studies in every pool and is attenuated when within-study variances are large, this finding is best interpreted as an absence of demonstrable statistical inconsistency rather than as evidence that the studies are exchangeable, and none of our conclusions rest on statistical homogeneity as a marker of clinical equivalence [18,19].

## 5. Conclusions

CMD is a common modality-dependent finding in patients undergoing TAVI for severe aortic stenosis. Our findings suggest that there is a post-TAVI increase in the CFR due to hemodynamic unloading (i.e., a reduced resting demand following afterload relief) rather than microvascular repair. A directionally consistent but quantitatively unstable prognostic association between CMD and MACE was observed, and the pooled HR should be regarded as hypothesis-generating only. There is an urgent need to standardize CMD assessment tools (i.e., indices, thresholds, and time points) to conduct adequately powered multicenter prognostic studies and reliable trials of post-TAVI CMD-targeted therapies.

### Impact on daily practice

CMD is present in roughly 4 in 10–1 in 2 patients undergoing TAVI, depending on whether wire-based or angiography-derived indices are used. Operators should not interpret the post-TAVI rise in CFR as evidence that the microcirculation has been repaired; minimal microvascular resistance, measured directly, is unchanged. CMD identified at the time of TAVI workup is directionally associated with worse MACE, but heterogeneity in cut-points and hazard ratio derivation across centers means the magnitude is not yet portable between cohorts. Until a consensus definition is established, centers reporting TAVI-CMD outcomes should always state the index used, the threshold applied, and whether the cut-point was prespecified or post hoc—without that information, prevalence and risk estimates from different cohorts cannot be meaningfully compared.

## Supporting information

S1 Table Database search strategies. All searches were performed in December 2025. The final included study was identified during a search of the reference list of Yones et al. [22].
(DOCX)

S2 Table PRISMA 2020 checklist (abbreviated). (DOCX)

S3 Table Detailed extraction characteristics of the included studies. (DOCX)

S4 Table Risk of bias determination using the Newcastle–Ottawa Scale (per study, full breakdown). Domains: Selection (S1–S4), Comparability (C1), and Outcome (O1–O3). Star = 1 (low risk for that domain). Maximum = 9.
(DOCX)

S5 Table Grading of Recommendations, Assessment, Development, and Evaluations (GRADE) evidence profile per outcome. (DOCX)

S6 Table Cohort overlap mapping and decisions. (DOCX)

S7 Table Coronary microvascular dysfunction diagnostic modalities and threshold definitions. (DOCX)

S8 Table Per-study extracted coronary flow reserve (CFR) and resistance values. Used for the CFR-change forest plot (Fig 3) and the resistance-change standardized mean difference pool (Fig 4).
(DOCX)

S9 Table Sensitivity analyses of all primary forest plots. (DOCX)

S10 Table Reasons for exclusion during full-text review. Of the 31 studies that passed the title and abstract screening, 19 were excluded during the full-text review.
(DOCX)
