# Supplementary material for: Coronary microvascular dysfunction in patients undergoing transcatheter aortic valve implantation: A systematic review and meta-analysis
Source: PLoS One. 2026 Oct 15;21(10):e0360374. doi: 10.1371/journal.pone.0360374 (PMC13671555; doi:10.1371/journal.pone.0360374)
Supplement: Supplementary file 1 [file pone.0360374.s001.docx]

**S1 Table. Database search strategies.**

All searches were performed in December 2025. The final included study was identified during a search of the reference list of Yones et al. (22).

| **Database** | **Records** | **Strategy** |
| --- | --- | --- |
| MEDLINE (PubMed) | 312 | ("transcatheter aortic valve replacement"[MeSH] OR "transcatheter aortic valve implantation"[tw] OR TAVI[tw] OR TAVR[tw]) AND ("coronary microvascular dysfunction"[tw] OR "microvascular resistance"[tw] OR "coronary flow reserve"[tw] OR "index of microcirculatory resistance"[tw] OR IMR[tw] OR MRR[tw] OR "microvascular resistance reserve"[tw] OR AMR[tw] OR IMRangio[tw] OR HMR[tw] OR CFVR[tw]) AND ("2010"[Date - Publication]:"2025"[Date - Publication]) |
| Embase (Ovid) | 198 | (transcatheter aortic valve implantation/exp OR TAVI.tw. OR TAVR.tw.) AND (coronary microvascular dysfunction.tw. OR microvascular resistance.tw. OR coronary flow reserve.tw. OR IMR.tw. OR MRR.tw. OR AMR.tw.) AND (2010:2025).pd. |
| Cochrane CENTRAL | 47 | (transcatheter aortic valve OR TAVI OR TAVR) AND (microvascular OR "flow reserve" OR IMR OR MRR OR AMR) |
| Web of Science | 63 | TS=((TAVI OR TAVR OR "transcatheter aortic valve") AND ("microvascular dysfunction" OR "flow reserve" OR IMR OR MRR OR AMR)). Indexes=SCI-EXPANDED. Timespan=2010-2025. |
| Scopus | 88 | TITLE-ABS-KEY((TAVI OR TAVR OR "transcatheter aortic valve") AND ("microvascular" OR "flow reserve" OR IMR OR MRR OR AMR)) AND PUBYEAR > 2009 AND PUBYEAR < 2026 |
| Grey literature | 31 | ClinicalTrials.gov, WHO ICTRP, ACC/ESC/TCT/EuroPCR proceedings 2023–2025, hand-search of reference lists |
| **TOTAL identified** | **708** | **After deduplication: 389 unique records** |
