# Supplementary material for: Coronary microvascular dysfunction in patients undergoing transcatheter aortic valve implantation: A systematic review and meta-analysis
Source: PLoS One. 2026 Oct 15;21(10):e0360374. doi: 10.1371/journal.pone.0360374 (PMC13671555; doi:10.1371/journal.pone.0360374)
Supplement: Supplementary file 2 [file pone.0360374.s002.docx]

# S2 Table. PRISMA 2020 Checklist (abbreviated).

| **Item #** | **Topic** | **Reported** | **Location** |
| --- | --- | --- | --- |
| 1 | Title — identify as systematic review | Yes | Title page |
| 2 | Structured abstract | Yes | Abstract section |
| 3 | Rationale | Yes | Section 1 (Introduction) |
| 4 | Objectives | Yes | Section 1, last paragraph |
| 5 | Eligibility criteria | Yes | Section 2.2 |
| 6 | Information sources | Yes | Section 2.3; Table S1 |
| 7 | Search strategy | Yes | Table S1 (full) |
| 8 | Selection process | Yes | Section 2.4 |
| 9 | Data collection process | Yes | Section 2.4 |
| 10 | Data items | Yes | Table S3 |
| 11 | Risk of bias assessment | Yes | Section 2.5; Table S4 |
| 12 | Effect measures | Yes | Sections 2.6.1–2.6.4 |
| 13 | Synthesis methods | Yes | Section 2.6 |
| 14 | Reporting bias assessment | Yes | Section 2.6.6 |
| 15 | Certainty assessment (GRADE) | Yes | Section 2.5; Table 5; Table S5 |
| 16 | Study selection (PRISMA flow) | Yes | Figure 1 |
| 17 | Study characteristics | Yes | Table 1; Table S3 |
| 18 | Risk of bias in studies | Yes | Table 3; Table S4 |
| 19 | Results of individual studies | Yes | Figures 2–5; Table S8 |
| 20 | Results of syntheses | Yes | Section 3; Table 4 |
| 21 | Reporting bias | Partial | Section 4.5 (limited by k < 10 in most pools) |
| 22 | Certainty of evidence | Yes | Table 5; Table S5 |
| 23 | Discussion (interpretation) | Yes | Section 4 |
| 24 | Registration and protocol | Yes | Title page; Section 2.1 |
| 25 | Support / funding | Yes | Title page |
| 26 | Competing interests | Yes | Title page |
| 27 | Availability of data, code, materials | Yes | All extracted data in Table S8; analysis code available on request |
