# Supplementary material for: Coronary microvascular dysfunction in patients undergoing transcatheter aortic valve implantation: A systematic review and meta-analysis
Source: PLoS One. 2026 Oct 15;21(10):e0360374. doi: 10.1371/journal.pone.0360374 (PMC13671555; doi:10.1371/journal.pone.0360374)
Supplement: Supplementary file 3 [file pone.0360374.s003.docx]

**S3 Table. Detailed extraction characteristics of the included studies.**

| **Study** | **PMID** | **Center/Country** | **Enrollment Period** | **CMD Modality** | **Notes** |
| --- | --- | --- | --- | --- | --- |
| Scarsini 2024a | 38040448 | Verona, Italy | 2019–2021 | IMRangio | Verona Valvular Heart Disease Registry; long-term prognostic cohort. |
| Scarsini 2024b | 37982178 | Verona/Aalst/Milan | 2021–2023 | MRR | Multicentre prospective; CMD = lowest MRR tertile (cohort-relative). |
| Shi 2025a | 40028840 | Fuwai, China | Sep 2012–Dec 2021 | AMR | Single-center Fuwai TAVR cohort; HR for MACE 1.94 [1.34, 2.81]. |
| Shi 2025b | 40254143 | Fuwai, China | 2012–2021 | AMRr (resting) | Same Fuwai cohort, asymptomatic/minimally symptomatic phenotype; only continuous HR available. |
| Gallinoro 2024 | 39374094 | Aalst, Belgium | Jun 2021–Jan 2023 | Q, Rμ, CFR (continuous thermo.) | 6-month follow-up subset n=20/51; absolute flow & resistance. |
| Gallinoro 2025 [sens] | 40211959 | 3 centers pooled | 2021–2023 | MRR (pooled) | Sensitivity-only; explicitly pooled Scarsini 2024b + 2 cohorts. |
| Gutiérrez-Barrios 2024 | 38232809 | Cádiz, Spain | Jun 2020–Jun 2021 | CFR, MRR (continuous thermo.) | 23 AS patients; 11 with 6-month follow-up; matched controls (n=17). |
| Minten 2025 | 39474985 | Belgium MC | Multicenter | IMR, MRR, CFR | Mixed AVR (68 TAVR / 38 SAVR); IMR not separately reported by AVR mode. |
| Wiegerinck 2015 | 26245891 | Amsterdam, NL | Pre-2015 | CFVR, HMR (Doppler) | ComboWire dual sensor; AR-free post-TAVI subset n=20. |
| Fezzi 2023 | 37712285 | Verona/CN | Retrospective | AMR; IMR ≥25 reference | Possible Verona patient overlap with Scarsini cohorts. |
| Sabbah 2023 | 36239118 | Copenhagen, Denmark | Two centers | CFR, Rμ (continuous thermo.) + CMR | Mixed AVR (17 TAVI / 14 SAVR-bio / 3 SAVR-mech); CFR not separately reported by AVR mode. |
| Yones 2025 | (in press) | Sheffield, UK | 2024–2025 | CFR, IMR, MVR (CMR) | Pilot; 7 patients (10 vessels); all had CAD requiring PCI. |

AMR=angiographic microvascular resistance; AMRr=resting angiographic microvascular resistance; AS=aortic stenosis; AVR=aortic valve replacement; bio=bioprosthesis; CAD=coronary artery disease; CFR=coronary flow reserve; CFVR=coronary flow velocity reserve; CMD=coronary microvascular dysfunction; CMR=cardiovascular magnetic resonance; HMR=hyperaemic microvascular resistance; IMR=index of microcirculatory resistance; IMRangio=angiography-derived index of microcirculatory resistance; MC=multicenter; mech=mechanical; MRR=microvascular resistance reserve; MVR=microvascular resistance; PCI=percutaneous coronary intervention; PMID=PubMed ID; Prosp.=prospective; Q=absolute coronary flow; Retro.=retrospective; Rμ=absolute minimal microvascular resistance; SAVR=surgical aortic valve replacement; TAVI=transcatheter aortic valve implantation; TAVR=transcatheter aortic valve replacement
