# Supplementary material for: Coronary microvascular dysfunction in patients undergoing transcatheter aortic valve implantation: A systematic review and meta-analysis
Source: PLoS One. 2026 Oct 15;21(10):e0360374. doi: 10.1371/journal.pone.0360374 (PMC13671555; doi:10.1371/journal.pone.0360374)
Supplement: Supplementary file 4 [file pone.0360374.s004.docx]

**S4 Table. Risk of bias determination using the Newcastle–Ottawa Scale (per study, full breakdown).**

Domains: Selection (S1–S4), Comparability (C1), and Outcome (O1–O3). Star = 1 (low risk for that domain). Maximum = 9.

| **Study** | **S1** | **S2** | **S3** | **S4** | **C1** | **O1** | **O2** | **O3** | **Total** | **Risk of Bias** |
| --- | --- | --- | --- | --- | --- | --- | --- | --- | --- | --- |
| Scarsini 2024a | ★ | ★ | ★ | ★ | ★★ | ★ | ★ | ★ | 9 | Low |
| Scarsini 2024b | ★ | ★ | ★ | ★ | ★★ | ★ | — | ★ | 8 | Low |
| Shi 2025a | ★ | ★ | ★ | ★ | ★★ | ★ | ★ | ★ | 9 | Low |
| Shi 2025b | ★ | ★ | — | ★ | ★ | ★ | ★ | ★ | 7 | Low |
| Gallinoro 2024 | ★ | ★ | ★ | ★ | ★★ | ★ | — | ★ | 8 | Low |
| Gallinoro 2025 [sens] | ★ | ★ | ★ | ★ | ★★ | ★ | — | ★ | 8 | Low |
| Gutiérrez-Barrios 2024 | ★ | — | ★ | ★ | ★ | ★ | — | ★ | 6 | Moderate |
| Wiegerinck 2015 | ★ | — | ★ | ★ | ★ | ★ | — | ★ | 6 | Moderate |
| Sabbah 2023 | ★ | — | ★ | ★ | ★★ | ★ | — | ★ | 7 | Low |
| Fezzi 2023 | ★ | ★ | ★ | ★ | ★★ | ★ | — | ★ | 8 | Low |
| Minten 2025 | ★ | ★ | ★ | ★ | ★★ | ★ | ★ | ★ | 9 | Low |
| Yones 2025 | ★ | — | — | ★ | ★ | ★ | — | ★ | 5 | Moderate |

Domains: S1=Representativeness; S2=Selection of non-exposed; S3=Ascertainment of exposure; S4=Outcome not present at start; C1=Comparability (max 2 stars); O1=Outcome assessment; O2=Adequate follow-up duration; O3=Adequacy of follow-up.
