# Supplementary material for: Coronary microvascular dysfunction in patients undergoing transcatheter aortic valve implantation: A systematic review and meta-analysis
Source: PLoS One. 2026 Oct 15;21(10):e0360374. doi: 10.1371/journal.pone.0360374 (PMC13671555; doi:10.1371/journal.pone.0360374)
Supplement: Supplementary file 5 [file pone.0360374.s005.docx]

**S5 Table. Grading of Recommendations, Assessment, Development, and Evaluations (GRADE) evidence profile per outcome.**

| **Outcome** | **Number**  **of**  **Studies** | **Design** | **Risk of Bias** | **Inconsistency** | **Indirect** | **Imprecise** | **Certainty (final)** |
| --- | --- | --- | --- | --- | --- | --- | --- |
| CMD prevalence — wire-based | 6 | Obs. cohort | Not serious | Serious (I²=72%) | Serious (heterogeneous thresholds) | Serious | ⊕ Very Low |
| CMD prevalence — angiographic | 4 | Obs. cohort | Not serious | Not serious (I²=0%) | Serious (centre-specific thresholds) | Not serious | ⊕⊕ Low |
| Δ CFR after TAVI | 5 | Before–after | Not serious | Serious (I²=72%) | Serious (no concurrent control) | Not serious | ⊕⊕ Low |
| Δ Microvascular resistance (SMD) | 4 | Before–after | Not serious | Not serious (I²=0%) | Very serious (incommensurable indices) | Serious (small N=85) | ⊕ Very Low |
| Prognostic HR for MACE | 2 | Obs. cohort | Not serious | Very serious (I²=78%) | Very serious (different CMD definitions) | Very serious (k=2; CI crosses unity) | ⊕ Very Low |

Certainty grading: ⊕⊕⊕⊕ High; ⊕⊕⊕ Moderate; ⊕⊕ Low; ⊕ Very Low. Observational designs start at Low and may be downgraded for risk of bias, inconsistency, indirectness, imprecision, and publication bias.

CFR=coronary flow reserve; CMD=coronary microvascular dysfunction; HR=hazard ratio; MACE=major adverse cardiovascular events; SMD=standardized mean difference; TAVI=transcatheter aortic valve implantation
