# Supplementary material for: Coronary microvascular dysfunction in patients undergoing transcatheter aortic valve implantation: A systematic review and meta-analysis
Source: PLoS One. 2026 Oct 15;21(10):e0360374. doi: 10.1371/journal.pone.0360374 (PMC13671555; doi:10.1371/journal.pone.0360374)
Supplement: Supplementary file 6 [file pone.0360374.s006.docx]

**S6 Table. Cohort overlap mapping and decisions.**

| **Pair / Issue** | **Overlap Type** | **Overlap Level** | **Decision and Sensitivity Test** |
| --- | --- | --- | --- |
| Scarsini 2024b ↔ Gallinoro 2025 | Explicit pooled re-analysis | HIGH | Gallinoro 2025 EXCLUDED from all primary forest plots; retained for sensitivity only. Sensitivity (with Gallinoro 2025): wire-based prevalence 46.0% vs primary 46.8%; CFR change MD +0.41 vs +0.49; SMD −0.29 vs −0.31. All within 1 SE of primary. |
| Scarsini 2024b ↔ Scarsini 2023 | Confirmed subset | HIGH | Scarsini 2023 was excluded from all meta-analyses (LFLG-AS subset of Scarsini 2024b); discussed narratively only. No sensitivity test (subset definitionally excluded). |
| Shi 2025a ↔ Shi 2025b | Shared registry, different phenotype | MODERATE | Both retained — different patient phenotype (symptomatic vs asymptomatic) and different CMD metrics (hyperemic AMR vs resting AMRr). Sensitivity excluding Shi 2025b: angiographic-based prevalence 38.0% vs primary 38.4%. |
| Scarsini 2024a ↔ Scarsini 2024b | Same center, different periods | LOW | Different modalities (IMRangio vs MRR); different cohorts confirmed independence by the authors. |
| Sabbah 2023 design | Mixed AVR (TAVI vs SAVR) | Design issue | TAVI subset (n=17) was used for the prevalence pool. EXCLUDED from the CFR-change pool because TAVI-specific paired CFR is not separately reported. Retained in the resistance SMD pool (full cohort n=34) because Rμ change is reported for the full cohort and is consistent across AVR modes. |
| Minten 2025 design | Mixed AVR (TAVR vs SAVR) | Design issue | The full cohort (n=116) was retained in the wire-based prevalence pool because IMR-defined CMD prevalence is reported for the combined cohort only. Sensitivity excluding Minten: wire-based prevalence 51.5% vs primary 46.8%, I² rises to 78%. |
| Verona axis (Scarsini × Fezzi) | Possible patient overlap | MODERATE | Sensitivity excluding Fezzi 2023 yielded an angiographic-based prevalence of 38.8% vs. primary 38.4% (negligible). Senior author overlap is acknowledged as a potential reproducibility-inflating factor. |
| Shi 2025b prognostic HR | Source HR not dichotomous | Methodological | EXCLUDED from the prognostic forest plot (Fig 5). Source publication reports continuous-scale HR (1.18 per 100u AMRr; CI 1.04–1.34) and event rates (42.6% vs 26.8%, p=0.042). Reported narratively in Section 3.6. |

AMR=angiographic microvascular resistance; AMRr=resting angiographic microvascular resistance; AVR=aortic valve replacement; CFR=coronary flow reserve; CI=confidence interval; CMD=coronary microvascular dysfunction; HR=hazard ratio; IMRangio=angiography-derived index of microcirculatory resistance; LFLG-AS=low-flow low-gradient aortic stenosis; MD=mean difference; MRR=microvascular resistance reserve; Rμ=absolute minimal microvascular resistance; SAVR=surgical aortic valve replacement; SE=standard error; SMD=standardized mean difference; TAVI=transcatheter aortic valve implantation; TAVR=transcatheter aortic valve replacement
