# Supplementary material for: Coronary microvascular dysfunction in patients undergoing transcatheter aortic valve implantation: A systematic review and meta-analysis
Source: PLoS One. 2026 Oct 15;21(10):e0360374. doi: 10.1371/journal.pone.0360374 (PMC13671555; doi:10.1371/journal.pone.0360374)
Supplement: Supplementary file 7 [file pone.0360374.s007.docx]

**S7 Table. Coronary microvascular dysfunction diagnostic modalities and threshold definitions.**

| **Modality** | **Index** | **Threshold (study-specific)** | **Biological Construct** |
| --- | --- | --- | --- |
| Wire-based bolus thermodilution | CFR (coronary flow reserve) | <2.0 (Wiegerinck, Gutiérrez-Barrios); <2.5 (Sabbah, Yones) | Ratio of hyperaemic to resting flow; integrates epicardial + microvascular physiology; sensitive to resting flow conditions. |
| Wire-based bolus thermodilution | IMR (index of microcirculatory resistance) | >25 (Minten, Yones) | Pd × mean transit time at hyperaemia; specific to microcirculation; depends on hyperaemic state achievement. |
| Wire-based continuous thermodilution | MRR (microvascular resistance reserve) | Lowest tertile (Scarsini 2024b); ≤3 (Gallinoro 2025; Minten) | CFR ÷ FFR with pressure correction; specific to microcirculation, independent of myocardial mass and driving pressures. |
| Doppler | CFVR / HMR | CFVR <2.0; HMR > control mean | Flow velocity-based; older methodology; valid but operator-dependent. |
| Angiography-derived | IMRangio | ≥30 (Scarsini 2024a) | Computational-flow-model surrogate for IMR; centre-derived threshold. |
| Angiography-derived (Murray's law) | AMR (hyperaemic) | >250 (Shi 2025a; Fezzi 2023 reference IMR ≥25) | Single-frame angiography + Murray's law; ROC-derived threshold from Fuwai cohort. |
| Angiography-derived (Murray's law) | AMRr (resting) | >490 (Shi 2025b) | Resting variant of AMR; no adenosine required; ROC-derived asymptomatic-AS-specific threshold. |
| Stress CMR | MVR (CMR-derived) | Various (Yones 2025: integrated with IMR) | Non-invasive stress perfusion; vessel-specific via computational model. |

AMR=angiographic microvascular resistance; AMRr=resting angiographic microvascular resistance; AS=aortic stenosis; CFR=coronary flow reserve; CFVR=coronary flow velocity reserve; CMD=coronary microvascular dysfunction; CMR=cardiovascular magnetic resonance; FFR=fractional flow reserve; HMR=hyperaemic microvascular resistance; IMR=index of microcirculatory resistance; IMRangio=angiography-derived index of microcirculatory resistance; MRR=microvascular resistance reserve; MVR=microvascular resistance; ROC=receiver operating characteristic
