# Supplementary material for: Coronary microvascular dysfunction in patients undergoing transcatheter aortic valve implantation: A systematic review and meta-analysis
Source: PLoS One. 2026 Oct 15;21(10):e0360374. doi: 10.1371/journal.pone.0360374 (PMC13671555; doi:10.1371/journal.pone.0360374)
Supplement: Supplementary file 8 [file pone.0360374.s008.docx]

**S8 Table. Per-study extracted coronary flow reserve (CFR) and resistance values.**

Used for the CFR-change forest plot (Fig 3) and the resistance-change standardized mean difference pool (Fig 4).

| **Study** | **n (paired)** | **CFR pre** | **CFR post** | **MD (CFR)** | **Resistance index** | **Δ Resistance** | **In Fig 3?** | **In Fig 4?** |
| --- | --- | --- | --- | --- | --- | --- | --- | --- |
| Wiegerinck 2015 | 20 | 1.90 ± 0.40 | 2.20 ± 0.60 | +0.30 | HMR (mmHg·cm·s⁻¹) | 2.03 → 1.66 | Yes | Yes |
| Gutiérrez-Barrios 2024 | 11 | 1.70 ± 0.40 | 2.30 ± 0.30 | +0.60 | MRR (×−1) | 1.95 → 2.67 | Yes | Yes |
| Gallinoro 2024 | 20 | 2.10 ± 0.50 | 2.30 ± 0.50 | +0.20 | Rμ (Wood units) | 0.56 → 0.42 | Yes | Yes |
| Scarsini 2024b† | 45 | 1.85 ± 1.36 | 2.50 ± 1.13 | +0.65 | MRR (lowest tertile) | (per-tertile data) | Yes | No |
| Yones 2025† | 7 | 1.53 ± 0.37 | 2.35 ± 0.52 | +0.82 | MVR (CMR) | Unchanged | Yes | No |
| Sabbah 2023* | 34 (full cohort) | med 2.5 [1.5–3.3] | med 3.1 [2.2–5.1] | Not extractable for TAVI subset | Rμ (mmHg·min/L) | 347 → 287 (NS) | No (excluded) | Yes (full cohort) |
| Minten 2025 | 116 (mixed AVR) | Not separately reported by AVR mode | — | — | IMR | All MV improved | No | No |

† Mean ± SD imputed from median [IQR] using Wan's method (Wan et al., BMC Med Res Methodol. 2014;14:135).

* Sabbah 2023: TAVI-specific paired CFR not reported in source; excluded from Fig 3. Full-cohort Rμ retained in Fig 4. with limitation flagged.

AVR=aortic valve replacement; CFR=coronary flow reserve; CMR=cardiovascular magnetic resonance; HMR=hyperaemic microvascular resistance; IMR=index of microcirculatory resistance; IQR=interquartile range; MD=mean difference; MRR=microvascular resistance reserve; MVR=microvascular resistance; NS=not significant; Rμ=absolute minimal microvascular resistance; SD=standard deviation; SMD=standardized mean difference; TAVI=transcatheter aortic valve implantation
