# Supplementary material for: Coronary microvascular dysfunction in patients undergoing transcatheter aortic valve implantation: A systematic review and meta-analysis
Source: PLoS One. 2026 Oct 15;21(10):e0360374. doi: 10.1371/journal.pone.0360374 (PMC13671555; doi:10.1371/journal.pone.0360374)
Supplement: Supplementary file 9 [file pone.0360374.s009.docx]

**S9 Table. Sensitivity analyses of all primary forest plots.**

| **Sensitivity Test** | **Primary Estimate** | **Sensitivity Estimate** | **Direction / Interpretation** |
| --- | --- | --- | --- |
| **Wire-based prevalence** | 46.8% [34.0–59.9], I²=72% | — | — |
| …with Gallinoro 2025 added | 46.8% | 46.0% [34.6–58.0], I²=68% | Stable; Gallinoro 2025 reduces I² slightly. |
| …excluding Yones 2025 (n=7, all impaired) | 46.8% | 43.9% [32.5–55.9], I²=56% | Heterogeneity falls; primary estimate stable. |
| …excluding Minten 2025 (mixed AVR) | 46.8% | 51.5% [37.0–65.6], I²=78% | Estimate shifts upward and I² increases; mixed-AVR design is a downward influence on heterogeneity. |
| **Angiographic prevalence** | 38.4% [35.3–41.7], I²=0% | — | — |
| …excluding Fezzi 2023 (Verona overlap) | 38.4% | 38.8% [35.4–42.2], I²=0% | Negligible change. |
| …excluding Shi 2025b (Fuwai overlap) | 38.4% | 38.0% [34.4–41.7], I²=0% | Negligible change. |
| **CFR change after TAVI** | MD +0.49 [+0.27, +0.71], I²=72% | — | — |
| …with Gallinoro 2025 added | MD +0.49 | MD +0.41 [+0.25, +0.57], I²=64% | Direction confirmed; absolute MD attenuated. |
| …excluding Yones 2025 (n=7, extreme effect) | MD +0.49 | MD +0.42 [+0.20, +0.64], I²=68% | Direction confirmed; primary remains within sensitivity envelope. |
| …excluding Scarsini 2024b (Wan-imputed) | MD +0.49 | MD +0.42 [+0.20, +0.64], I²=70% | Direction confirmed; Wan-imputation does not drive estimate. |
| **Microvascular resistance SMD** | SMD −0.31 [−0.53, −0.09], I²=0% | — | — |
| …with Gallinoro 2025 added | SMD −0.31 | SMD −0.29 [−0.49, −0.09], I²=0% | Direction confirmed. |
| …excluding Sabbah 2023 (mixed AVR) | SMD −0.31 | SMD −0.39 [−0.69, −0.09], I²=0% | Direction strengthens; CI widens with smaller N (n=51). |
| **Prognostic HR for MACE — exploratory** | HR 3.22 [0.98, 10.60], I²=78% | — | — |
| …Scarsini 2024a alone | HR 3.22 | HR 6.67 [2.25, 19.78] | Single-study estimate; informative only. |
| …Shi 2025a alone | HR 3.22 | HR 1.94 [1.34, 2.81] | Single-study estimate; informative only. The wide pooled CI is driven by between-study divergence, not by within-study uncertainty. |

AVR=aortic valve replacement; CFR=coronary flow reserve; CI=confidence interval; HR=hazard ratio; MACE=major adverse cardiovascular events; MD=mean difference; SMD=standardized mean difference; TAVI=transcatheter aortic valve implantation
