# Supplementary material for: Coronary microvascular dysfunction in patients undergoing transcatheter aortic valve implantation: A systematic review and meta-analysis
Source: PLoS One. 2026 Oct 15;21(10):e0360374. doi: 10.1371/journal.pone.0360374 (PMC13671555; doi:10.1371/journal.pone.0360374)
Supplement: Supplementary file 10 [file pone.0360374.s010.docx]

**S10 Table. Reasons for exclusion during full-text review.**

Of the 31 studies that passed the title and abstract screening, 19 were excluded during the full-text review.

| **Reason for Exclusion** | **# Studies** | **Examples / Notes** |
| --- | --- | --- |
| Insufficient TAVI-specific quantitative CMD data | 8 | Studies of CMD in AS without TAVI-specific subgroup analysis or pre/post-TAVI quantitative data extractable. |
| Conference abstract only | 4 | Insufficient methodological detail for quality appraisal and data extraction. |
| Confirmed cohort overlap (subset) | 3 | Scarsini 2023 (subset of Scarsini 2024b); 2 other excluded subsets. |
| Mixed AVR — TAVI not separately extractable | 2 | Studies enrolling SAVR predominantly or pooled AVR cohorts where TAVI-specific data could not be obtained. |
| Protocol only — no results | 1 | Dobrolinska 2022 (Biomimetics protocol paper). |
| Non-quantitative CMD assessment | 1 | Qualitative imaging assessment without index-based CMD definition. |

Two additional borderline studies (Zhou 2022 MIDAS, Alahdab 2024 JAHA) were considered but excluded because they were either cross-sectional CMD-in-AS studies without TAVI-specific outcomes or had AVR subgroups too small (n < 5) to extract.

AS=aortic stenosis; AVR=aortic valve replacement; CMD=coronary microvascular dysfunction; SAVR=surgical aortic valve replacement; TAVI=transcatheter aortic valve implantation
